# Transcriptional Activation of Matricellular Protein Spondin2 (SPON2) by BRG1 in Vascular Endothelial Cells Promotes Macrophage Chemotaxis

**DOI:** 10.3389/fcell.2020.00794

**Published:** 2020-08-14

**Authors:** Nan Li, Shuai Liu, Yuanyuan Zhang, Liming Yu, Yanjiang Hu, Teng Wu, Mingming Fang, Yong Xu

**Affiliations:** ^1^Department of Cardiothoracic Surgery, Liyang People’s Hospital, Liyang, China; ^2^Department of Clinical Medicine and Laboratory Center for Experimental Medicine, Jiangsu Health Vocational Institute, Nanjing, China; ^3^Key Laboratory of Targeted Intervention of Cardiovascular Disease and Collaborative Innovation Center for Cardiovascular Translational Medicine, Department of Pathophysiology, Nanjing Medical University, Nanjing, China; ^4^Institute of Biomedical Research, Liaocheng University, Liaocheng, China; ^5^Hainan Provincial Key Laboratory for Tropical Cardiovascular Diseases Research and Key Laboratory of Emergency and Trauma of Ministry of Education, Institute of Cardiovascular Research of the First Affiliated Hospital, Hainan Medical University, Haikou, China; ^6^Department of Cardiology, Kaifeng Central Hospital, Kaifeng, China

**Keywords:** transcriptional regulation, epigenetics, endothelial cells, macrophage, atherosclerosis

## Abstract

The matricellular protein SPON2 plays diverse roles in the development of cardiovascular diseases. SPON2 is expressed in endothelial cells, but its transcription regulation in the context of atherogenesis remains incompletely appreciated. Here we report that SPON2 expression was up-regulated by pro-atherogenic stimuli (oxLDL and TNF-α) in vascular endothelia cells. In addition, endothelial SPON2 was elevated in *Apoe*^–/–^ mice fed on a Western diet compared to the control mice. Induction of SPON2 in endothelial cells by pro-atherogenic stimuli was mediated by BRG1, a chromatin remodeling protein, both *in vitro* and *in vivo*. Further analysis revealed that BRG1 interacted with the sequence-specific transcription factor Egr-1 to activate SPON2 transcription. BRG1 contributed to SPON2 *trans*-activation by modulating chromatin structure surrounding the SPON2 promoter. Functionally, activation of SPON2 transcription by the Egr-1/BRG1 complex provided chemoattractive cues for macrophage trafficking. SPON2 depletion abrogated the ability of BRG1 or Egr-1 to stimulate endothelial derived chemoattractive cue for macrophage migration. On the contrary, recombinant SPON2 rescued endothelial chemo-attractability in the absence of BRG1 or Egr-1. In conclusion, our data have identified a novel transcriptional cascade in endothelial cells that may potentially promote macrophage recruitment and vascular inflammation leading to atherogenesis.

## Introduction

Atherosclerosis is a major form of coronary heart disease (CHD). Rupture of atherosclerotic plaques represents a major cause for acute myocardial infarction and sudden cardiac death (Schwartz et al., 2009; Hamesch et al., 2015; Fan et al., 2020). A host of risk factors, including smoking, obesity, and dyslipidemia, can contribute to atherogenesis (Feng et al., 2011; Miura et al., 2012). It is generally believed that atherosclerosis is a prototypical human pathology of chronic inflammation (Hansson and Libby, 2006). Accumulation of pro-inflammatory cells and mediators within the plaque collectively destabilizes the fibrous cap and renders the plaque prone to rupture. This notion, long since authenticated in model animals, has recently received evidentiary support from a large clinical trial involving over 10,000 enrolled patients: an antibody (Canakinumab) targeting the pro-inflammatory cytokine interleukin 1 beta (IL-1β) significantly lowered the rate of re-occurring cardiovascular events (Ridker et al., 2017).

During atherogenesis, pro-inflammatory immune cells gain access to the vasculature via a series of tightly regulated processes. The vascular cells produce and emit chemoattractive cues that stimulate the homing/trafficking of immune cells (Liu et al., 2012). For instance, it has been demonstrated that arterial colony stimulating factor (CSF) plays a key role promoting monocyte migration during atherogenesis (Shaposhnik et al., 2010). Smooth muscle cells can produce chemokine (C-X3-C motif) ligand 1 (CX3CL1) and C-C motif chemokine 22 (CCL22), in response to atherogenic lipids, to promote macrophage trafficking (Barlic et al., 2007; Kimura et al., 2018). Once the immune cells are recruited to the vessel wall, rolling on and adhesion to the endothelial layer are mediated by a group of adhesion proteins, including intercellular adhesion molecules (ICAMs), vascular cell adhesion molecules (VCAMs), and selectins, which can be up-regulated by pro-atherogenic stimuli on the surface of activated endothelial cells (He, 2010). In accordance, there is evidence that blockade of chemokine signaling or leukocyte-endothelial cell interaction can lead to attenuation of immune infiltrates in the plaque and atherosclerosis overall in animal models.

Spondin 2 (SPON2, also known as Mindin) is a member of the Mindin-F-Spondin family of matricellular proteins functioning as pattern recognition receptors to regulate immunity; SPON2 itself contains a C-terminal thrombospondin type 1 repeat (TSR) that mediates its binding to LPS to initiate the TLR signaling critical to innate immunity (Li et al., 2009). SPON2 is expressed in a variety of cell types and can participate in cell adhesion, migration, and differentiation (Feinstein et al., 1999; Zhu et al., 2015; Schmid et al., 2016; Zhang et al., 2018d). Of note, SPON2 expression is up-regulated in the arteries in *Apoe*^–/–^ mice fed with a Western diet to induce atherosclerosis compared to the control mice although the underlying mechanism remains unclear (Zhang et al., 2018a). BRG1, encoded by *SMARCA4*, is a component of the mammalian chromatin remodeling complex. BRG1 is involved in the pathogenesis of human diseases by regulating cell-type and context-specific transcriptional events (Xu and Fang, 2012; Chang and Han, 2016; Wu et al., 2017). Previously we have demonstrated that BRG1 activates the transcription of a slew of adhesion proteins in endothelial cells in response to pro-inflammatory stimuli (Fang et al., 2013). Congruently, endothelial-specific BRG1 deletion attenuates atherosclerosis in *Apoe*^–/–^ mice with decreased adhesion of immune cells to the vessel wall. Here we report that SPON2 expression can be up-regulated by pro-atherogenic stimuli in endothelial cells both *in vitro* and *in vivo* in a BRG1-dependent manner. BRG1 cooperates with Egr-1 to activate SPON2 transcription, which in turn promotes macrophage migration.

## Materials and Methods

### Animals

All the animal experiments were reviewed and approved by the intramural Ethics Committee on Humane Treatment of Experimental Animals. Endothelial-specific deletion of BRG1 was achieved by crossing the *Smarca4*^f/f^ strain (Li et al., 2018a, b) with the *Cdh5*-Cre strain (Li et al., 2018e) and the *Apoe^–/–^* strain. The breedings were conducted by Nanjing Biomedical Research Institute of Nanjing University. Atherosclerosis was induced by feeding with a Western diet (D12109, Research Diets) for 12 weeks.

### Cell Culture, Transient Transfection, and Reporter Assay

Immortalized human umbilical vein endothelial cells (EAhy926, ATCC) were maintained in DMEM supplemented with 10% FBS. Primary human aortic endothelial cells (HAECs, Lonza) were maintained in EGM-2 media with supplements supplied by the vendor; experiments were performed in primary cells between 3rd and 6^th^ passages (Li et al., 2018d; Zhang et al., 2018b). Primary murine peritoneal macrophages were isolated and cultured in DMEM supplemented with 10% FBS as previously described (Liu et al., 2018). Primary murine bone marrow derived macrophages (BMDM) were isolated and differentiated as previously described (Yu et al., 2014). Myc-tagged BRG1, FLAG-tagged EGR-1, and human SPON2 promoter-luciferase constructs have been described previously (Liao et al., 2010; Yu et al., 2017; Wang et al., 2018; Zhang et al., 2018c). Small interfering RNAs were purchased from Dharmacon. Transient transfection was performed with Lipofectamine 2000. Cells were harvested 48 h after transfection and reporter activity was measured using a luciferase reporter assay system (Promega) as previously described (Li et al., 2018c).

### RNA Isolation and Real-Time PCR

RNA was extracted with the RNeasy RNA isolation kit (Qiagen) as previously described (Yang et al., 2019a, b; Zhang et al., 2019). Reverse transcriptase reactions were performed as previously described using a SuperScript First-strand Synthesis System (Invitrogen) (Zeng et al., 2018). Real-time qPCR reactions were performed in triplicate wells on an ABI STEPONEPlus (Life Tech). The relative quantification for a given gene was normalized by the *Gapdh* mRNA values. All experiments were repeated three times in triplicate wells.

### Protein Extraction, Immunoprecipitation, and Western Blotting

Whole cell lysates were obtained by re-suspending cell pellets in RIPA buffer with freshly added protease inhibitor tablet (Roche). Immunoprecipitation was performed essentially as previously described (Yang et al., 2018; Shao et al., 2019; Weng et al., 2019). Briefly, anti-Brg1 (Santa Cruz, sc-17796), anti-Egr-1 (Abcam, ab55160), or pre-immune IgGs (P.I.I.) were added to and incubated with cell lysates overnight before being absorbed by Protein A/G-plus Agarose beads (Santa Cruz). Precipitated immune complex was released by boiling with 1X SDS electrophoresis sample buffer. Western analyses were performed with anti-β-actin (Sigma, A2228), anti-Brg1 (Santa Cruz, sc-17796), anti-Egr-1 (Abcam, ab55160), or anti-SPON2 (Proteintech, 20513-1).

### Macrophage Migration Assay

Macrophage migration was measured using the Boyden chamber inserts (5 μm, Corning) as previously described (Jia et al., 2005). Briefly, primary peritoneal macrophages or BMDMs were added to the upper chamber along with specified conditioned media or recombinant Mindin (20 ng/ml, R&D) whereas complete DMEM media were added to the lower chamber. The number of migrated macrophages in the lower chamber was counted in five randomly chosen fields using an inverted microscope. All experiments were performed in triplicates and repeated three times.

### Chromatin Immunoprecipitation (ChIP)

ChIP assays were performed essentially as described before (Fan et al., 2019; Kong et al., 2019a, b; Li et al., 2019a, b, c, d, e; Liu L. et al., 2019; Lu et al., 2019; Shao et al., 2019; Weng et al., 2019; Yang et al., 2019a, b; Zhao et al., 2019; Dong et al., 2020; Fan et al., 2020; Lv et al., 2020; Mao et al., 2020a, b) with the following antibodies: anti-Brg1 (Santa Cruz, sc-17796), anti-histone H3 (Millipore, 06-755), anti-acetyl histone H3 (Millipore, 06-599), anti-trimethyl H3K4 (Millipore, 07-473), anti-dimethyl H3K9 (Millipore, 07-441), anti-Egr-1 (Thermo Fisher, MA5-15009), or IgG. All experiments were repeated three times in triplicate wells.

### Statistical Analysis

One-way ANOVA with *post hoc* Scheff’e analyses were performed by SPSS software (IBM SPSS v18.0, Chicago, IL, United States). *P*-values less than 0.05 were considered statistically significant.

## Results

### Up-Regulation of SPON2 Expression in Endothelial Cells by Pro-atherogenic Stimuli

**FIGURE 1:**
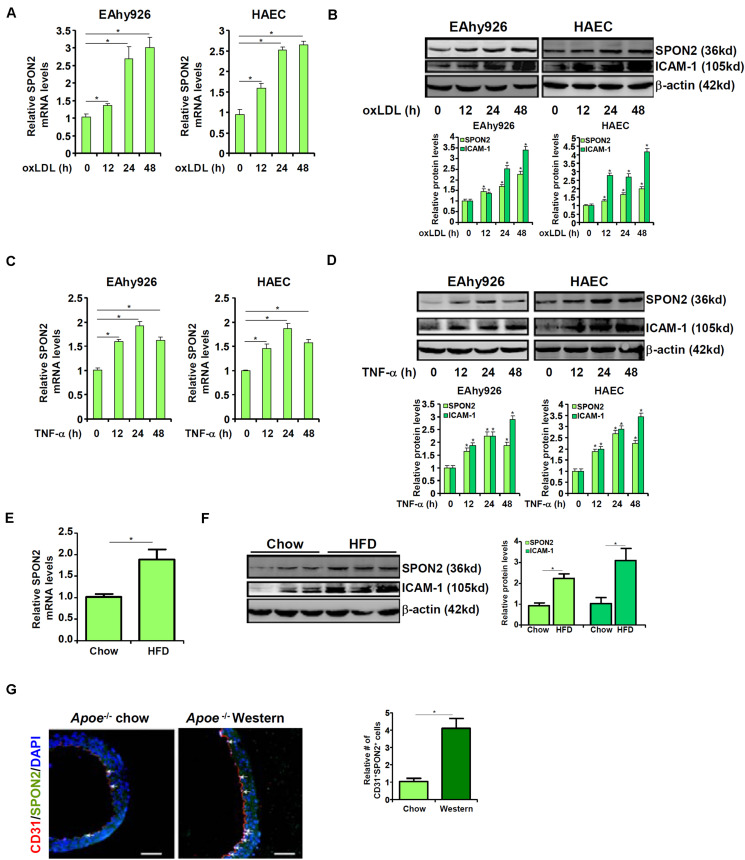
Up-regulation of SPON2 expression in endothelial cells by pro-atherogenic stimuli. **(A,B)** EAhy926 cells and HAECs were treated with or without oxLDL (50 μg/ml) and harvested at indicated time points. SPON2 expression was measured by qPCR and Western. **(C,D)** EAhy926 cells and HAECs were treated with or without TNF-α (10 ng/ml) and harvested at indicated time points. SPON2 expression was measured by qPCR and Western. **(E–G)** Apoe^– /–^ mice were fed with a high-fat diet (HFD) or a control diet (chow) for 12 weeks. SPON2 expression in the arteries was measured by qPCR, Western, and immunofluorescence staining. *p < 0.05.

We first examined whether SPON2 expression in vascular endothelial cells could be influenced by pro-atherogenic stimuli. To this end, immortalized human endothelial cells (EAhy926) and primary human aortic endothelial cells (HAECs) were treated with oxLDL, a well-documented risk factor for atherosclerosis (Toshima et al., 2000). SPON2 mRNA (Figure 1A) and protein (Figure 1B) levels were up-regulated by oxLDL treatment in both types of cells. Next, the cells were treated with TNF-α, another known risk marker for atherosclerosis (McKellar et al., 2009). Similar to oxLDL treatment, TNF-α treatment also stimulated the expression of SPON2 in endothelial cells at both mRNA (Figure 1C) and protein (Figure 1D) levels. We then examined the expression of SPON2 in arteries during atherogenesis in mice. *Apoe*^–/–^ mice were fed a Western diet for 12 weeks to induce atherosclerosis; significant atherosclerotic lesions were detected in these mice compared to those mice fed a control diet (Supplementary Figure S1). As shown in Figures 1E,F, SPON2 expression was significantly higher in the arteries of the atherosclerotic mice than the control mice. More important, endothelial SPON2 expression was up-regulated in the arteries of the atherosclerotic mice compared to the control mice as assessed by immunofluorescence staining of SPON2^+^CD31^+^ cells (Figure 1G).

### BRG1 Regulates SPON2 Expression in Endothelial Cells

**FIGURE 2:**
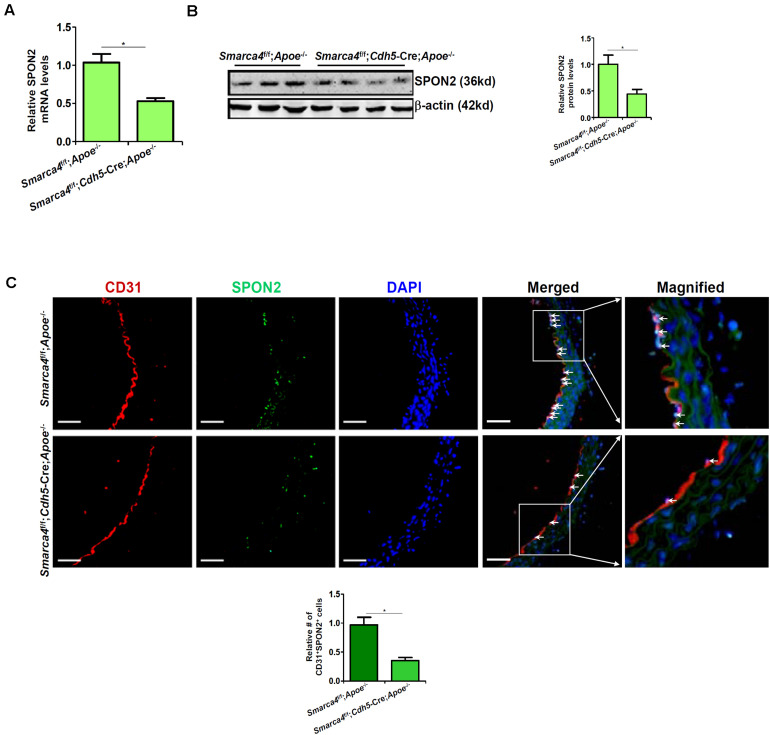
BRG1 regulates SPON2 expression in mice. **(A–C)** *Smarca4*^f/f^-*Apoe*^– /–^ mice and *Smarca4*^f/f^-*Cdh5*-Cre-*Apoe*^– /–^ mice were fed with an HFD for 12 weeks. SPON2 expression in the arteries was measured by qPCR, Western, and immunofluorescence staining. *p < 0.05.

We have previously shown that endothelial-specific depletion of BRG1 attenuates atherogenesis in mice (Fang et al., 2013). When endothelial conditional BRG1 knockout mice (*Smarca4*^f/f^; *Cdh5*-Cre) were crossed with the *Apoe*^–/–^ mice and placed on a Western diet, development of atherosclerotic lesions was significantly attenuated compared to the control mice (Supplementary Figure S2). Coincidently, quantitative PCR (Figure 2A), Western blotting (Figure 2B), and immunofluorescence staining of SPON2^+^CD31^+^ cells (Figure 2C) all showed that BRG1 deficiency in endothelial cells resulted in a decrease in SPON2 expression.

**FIGURE 3:**
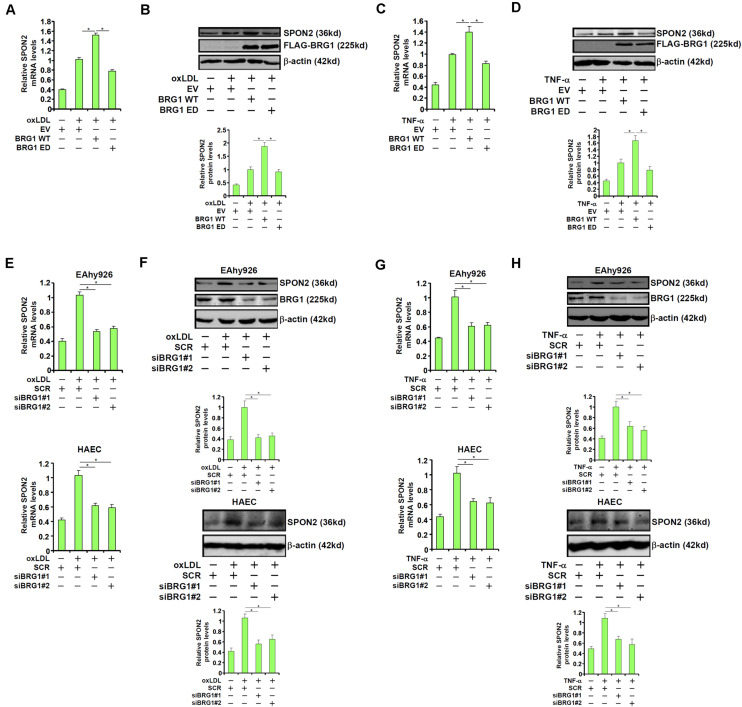
BRG1 regulates SPON2 expression in cultured endothelial cells. **(A,B)** EAhy926 cells were transfected with wild type (WT) or enzyme deficient (ED) BRG1 followed by treatment with oxLDL (50 μg/ml) for 24 h. SPON2 expression was measured by qPCR and Western. **(C,D)** EAhy926 cells were transfected with wild type (WT) or enzyme deficient (ED) BRG1 followed by treatment with TNF-α (10 ng/ml) for 24 h. SPON2 expression was measured by qPCR and Western. **(E,F)** EAhy926 cells or HAECs were transfected with siRNA targeting BRG1 or scrambled siRNA (SCR) followed by treatment with oxLDL (50 μg/ml) for 24 h. SPON2 expression was measured by qPCR and Western. **(G,H)** EAhy926 cells or HAECs were transfected with siRNA targeting BRG1 or scrambled siRNA (SCR) followed by treatment with TNF-α (10 ng/ml) for 24 h. SPON2 expression was measured by qPCR and Western. *p < 0.05.

We were prompted to investigated the possibility BRG1 may be essential for the regulation of SPON2 expression in response to pro-atherogenic stimuli. Over-expression of wild type (WT) BRG1, but not enzyme deficient (ED) BRG1, enhanced the induction of SPON2 expression in endothelial cells by oxLDL treatment (Figures 3A,B). In addition, BRG1 over-expression further augmented SPON2 induction by TNF-α (Figures 3C,D). On the contrary, BRG1 knockdown by two separate pairs of siRNAs (Supplementary Figure S3 for knockdown efficiencies) ameliorated the induction of SPON2 expression in endothelial cells by either oxLDL treatment (Figures 3E,F) or TNF-α treatment (Figures 3G,H). Of note, SPON2 depletion did not influence the expression levels of adhesion molecules ICAM-1 and VCAM-1, two documented BRG1 targets, in endothelial cells treated with either oxLDL (Supplementary Figure S4A) or TNF-α (Supplementary Figure S4B). Consistently, occupancies of BRG1 on the ICAM-1 promoter and the VCAM-1 promoter were not altered by SPON2 knockdown (Supplementary Figure S4C), suggesting that the relationship between BRG1 and SPON2 is not reciprocal but rather unidirectional.

### BRG1 Regulates Macrophage Trafficking Through SPON2

**FIGURE 4:**
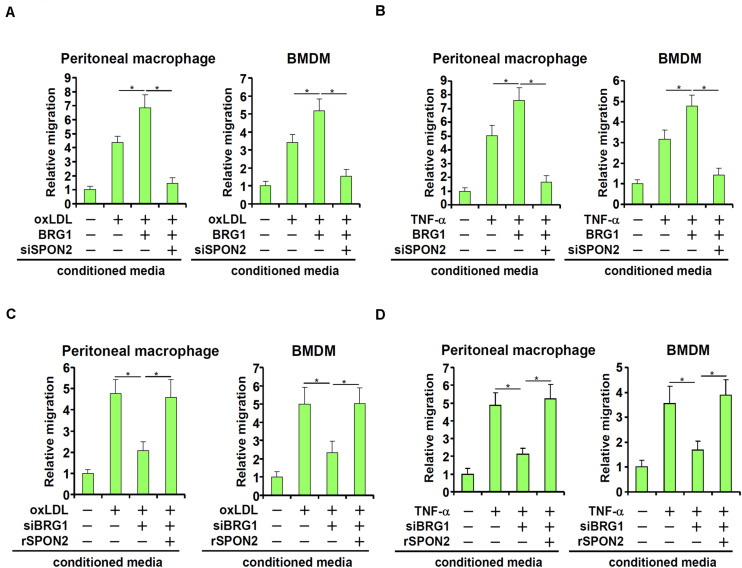
BRG1 regulates macrophage trafficking through SPON2. **(A)** EAhy926 cells were transfected with BRG1 and/or siRNA targeting SPON2 followed by treatment with oxLDL (50 μg/ml) for 24 h. Macrophage migration assay was performed and quantified as described in Section “Materials and Methods.” **(B)** EAhy926 cells were transfected with BRG1 and/or siRNA targeting SPON2 followed by treatment with TNF-α (10 ng/ml) for 24 h. Macrophage migration assay was performed and quantified as described in Methods. **(C)** EAhy926 cells were transfected with siRNA targeting BRG1 in the presence or absence of followed by treatment with oxLDL (50 μg/ml) for 24 h. Recombinant SPON2 was added to the supernatant. Macrophage migration assay was performed and quantified as described in Section “Materials and Methods.” **(D)** EAhy926 cells were transfected with siRNA targeting BRG1 in the presence or absence of followed by treatment with TNF-α (10 ng/ml) for 24 h. Recombinant SPON2 was added to the supernatant. Macrophage migration assay was performed and quantified as described in Section “Materials and Methods.” *p < 0.05.

SPON2 has a role in macrophage trafficking, a key process in atherogenesis. We decided to investigate the functional relevance of BRG1-mediated induction of SPON2 expression in endothelial cells. BRG1 was over-expressed in endothelial cells followed by treatment with oxLDL. Conditioned medium (CM) was collected and used as a chemoattractant to induce macrophage migration. As shown in Figure 4A, CM collected from oxLDL-treated endothelial cells exhibited stronger chemoattractive potency, which was further enhanced by BRG1 over-expression; SPON2 knockdown severely compromised the chemoattractive capability of the conditioned media. Likewise, TNF-α treatment stimulated the emission of a chemoattractive signal from the endothelial cells, which was further enhanced by BRG1 over-expression but blunted by SPON2 depletion (Figure 4B). On the other hand, BRG1 silencing blocked the production and release of a chemoattractive cue from oxLDL-treated endothelial cells, which could be rescued by the addition of recombinant SPON2 in the CM (Figure 4C). Finally, SPON2 supplementation recovered the deficiency in macrophage chemotaxis following BRG1 knockdown (Figure 4D). We also profiled the expression of several other chemokines. Treatment with either oxLDL (Supplementary Figure S5A) or TNF-α (Supplementary Figure S5B) markedly up-regulated the expression of CCL2/MCP-1, CCL3, and CCL5, which was further augmented by BRG1 over-expression. SPON2 depletion, however, did not appreciably impact the expression of these chemokines. These data collectively suggest that BRG1 activates the production of SPON2 in endothelial cells in response to pro-atherogenic stimuli to promote macrophage chemotaxis.

### BRG1 Directly Regulates SPON2 Transcription in Endothelial Cells

**FIGURE 5:**
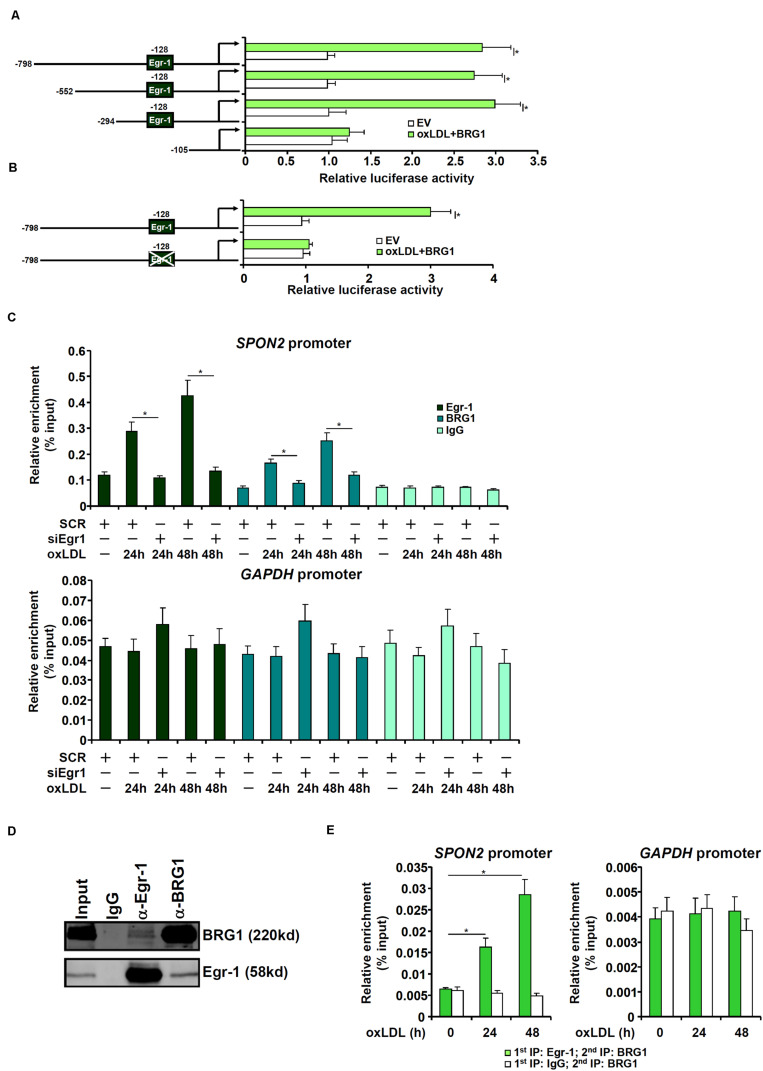
BRG1 directly regulates SPON2 transcription in endothelial cells. **(A)** SPON2 promoter-luciferase constructs of various lengths were transfected into EAh926 cells with or without BRG1 followed by treatment with oxLDL (50 μg/ml) for 24 h. Luciferase activities were normalized by both protein concentration and GFP fluorescence. **(B)** Wild type or mutant SPON2 promoter-luciferase construct was transfected into EAhy926 cells with or without BRG1 followed by treatment with oxLDL (50 μg/ml) for 24 h. Luciferase activities were normalized by both protein concentration and GFP fluorescence. **(C)** EAhy926 cells were transfected with siRNA targeting Egr-1 or SCR followed by treatment with oxLDL (50 μg/ml) for 24 h.ChIP assays were performed with indicated antibodies. Inset, knockdown efficiency of Egr-1 was examined by Western. **(D)** EAhy926 cells were treated with oxLDL (50 μg/ml) for 24 h. Nuclear proteins were extracted and immunoprecipitated with indicated antibodies. **(E)** EAhy926 cells were treated with or without oxLDL (50 μg/ml) for 24 h. Re-ChIP assays were performed with indicated antibodies. Data represent averages of three independent experiments and error bars represent SEM. *p < 0.05.

To examine whether activation of SPON2 expression by BRG1 occurred at the transcriptional level, human SPON2 promoter-luciferase constructs of different lengths were transfected into endothelial cells with or without BRG1. As shown in Figure 5A, over-expression of BRG1 activated the three longer SPON2 constructs but not the shortest SPON2 construct, from which a binding site for Egr-1 was missing, suggesting that Erg-1 might recruit BRG1 to the SPON2 promoter to regulate transcription. This Egr-1 motif was conserved in the human SPON2 promoter and the mouse SPON2 promoter (Supplementary Figure S6). Indeed, mutation this Egr-1 site within the SPON2 promoter completely abrogated activation by BRG1 over-expression (Figure 5B). Next, ChIP assays were performed to evaluate the binding of Egr-1 and BRG1 on the SPON2 promoter. When the endothelial cells were exposed to oxLDL, both Egr-1 and BRG1 were recruited to the SPON2 promoter, but not the GAPDH promoter at 24 and 48 h; depletion of Egr-1 suppressed the binding of both Egr-1 and BRG1 to the SPON2 promoter (Figure 5C). Co-immunoprecipitation assay confirmed that Egr-1 and BRG1 interacted with each other in endothelial cells (Figure 5D). More important, oxLDL treatment promoted the assembly of an Egr-1-BRG1 complex on the SPON2 promoter (Figure 5E).

### Egr-1 Mediates SPON2 Transcription to Regulate Macrophage Trafficking

**FIGURE 6:**
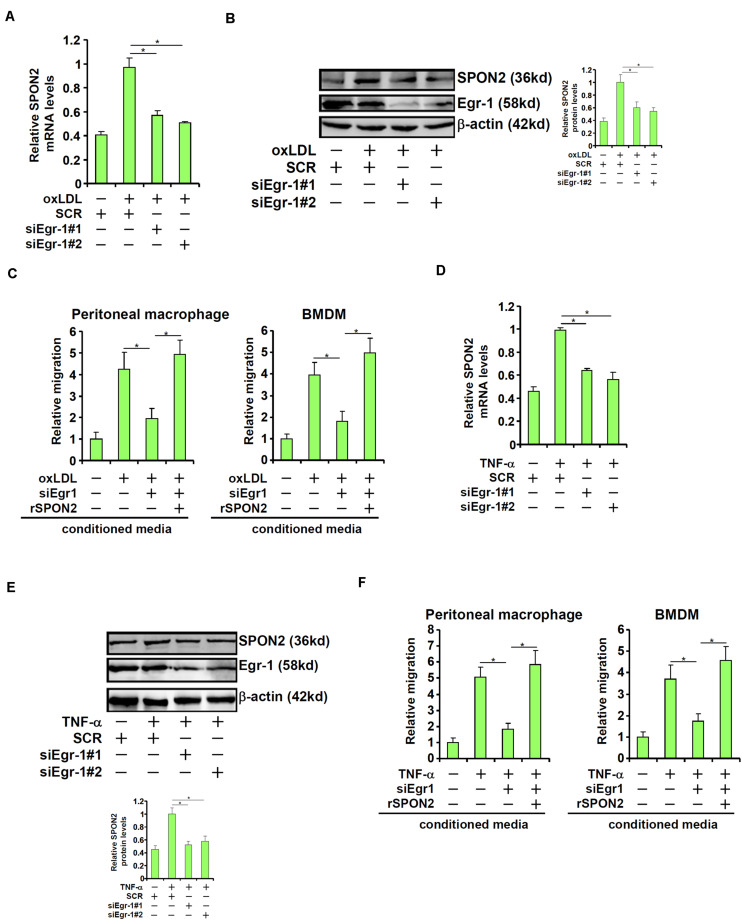
Egr-1 mediates SPON2 transcription to regulate macrophage trafficking. **(A–C)** EAhy926 cells were transfected with siRNA targeting Egr-1 or scrambled siRNA (SCR) followed by treatment with oxLDL (50 μg/ml) for 24 h. SPON2 expression was measured by qPCR and Western. Recombinant SPON2 was added to the supernatant. Macrophage migration assay was performed and quantified as described in Section “Materials and Methods.” **(D–F)** EAhy926 cells were transfected with siRNA targeting Egr-1 or scrambled siRNA (SCR) followed by treatment with TNF-α (10 ng/ml) for 24 h. SPON2 expression was measured by qPCR and Western. Recombinant SPON2 was added to the supernatant. Macrophage migration assay was performed and quantified as described in Section “Materials and Methods.” *p < 0.05.

Next, we investigated the role of Egr-1 in SPON2 *trans*-activation in endothelial cells. When Egr-1 was depleted with siRNAs, oxLDL-induced expression of SPON2 was significantly dampened (Figures 6A,B). As a functional readout, macrophage trafficking was evaluated using conditioned media collected from these cells as chemoattractive cues. Egr-1 knockdown suppressed macrophage migration induced by oxLDL and the addition of recombinant SPON2 restored macrophage migration (Figure 6C). Similarly, Egr-1 silencing blocked SPON2 induction by TNF-α treatment in endothelial cells (Figures 6D,E). Egr-1 deficiency in endothelial cells also blocked the migration of macrophages in response to the conditioned media, which could be mitigated by recombinant SPON2 (Figure 6F).

### BRG1 Contributes to SPON2 Transcription by Modulating Chromatin Structure

**FIGURE 7:**
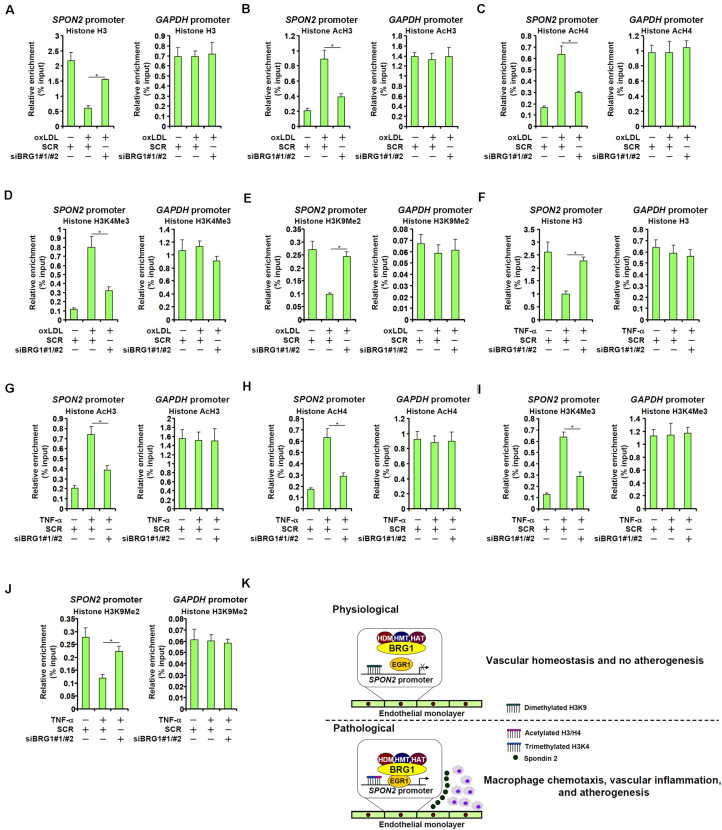
BRG1 contributes to SPON2 transcription by modulating chromatin structure. **(A–E)** EAhy926 cells were transfected with siRNA targeting BRG1 or scrambled siRNA (SCR) followed by treatment with oxLDL (50 μg/ml) for 24 h. ChIP assays were performed with anti-histone H3 **(A)**, anti-acetyl H3 **(B)**, anti-acetyl H4 **(C)**, anti-trimethyl H3K4 **(D)**, and anti-dimethyl H3K9 **(E)**. **(F–J)** EAhy926 cells were transfected with siRNA targeting BRG1 or scrambled siRNA (SCR) followed by treatment with TNF-α (10 ng/ml) for 24 h. ChIP assays were performed with anti-histone H3 **(F)**, anti-acetyl H3 **(G)**, anti-acetyl H4 **(H)**, anti-trimethyl H3K4 **(I)**, and anti-dimethyl H3K9 **(J)**. **(K)** A schematic model. *p < 0.05.

We finally examined the epigenetic mechanism by which BRG1 contributes to SPON2 *trans*-activation. Eviction of histones from the gene promoters driven by BRG1-mediated remodeling activity is considered a major mechanism for transcriptional activation (Jones et al., 2003; Hargreaves and Crabtree, 2011). ChIP assay showed that in response to oxLDL treatment, there were fewer histones wrapped around the SPON2 promoter, indicative of histone eviction and thus chromatin loosening; BRG1 depletion, however, normalized the association of histones with the SPON2 promoter (Figure 7A). In addition, BRG1 is known to interact with various histone modifying enzymes to influence gene expression (Tian et al., 2013; Weng et al., 2015; Xu et al., 2015; Zhang et al., 2018b, c, 2019; Li et al., 2019e). It was observed that histone markers associated with active chromatin, including acetyl H3 (Figure 7B), acetyl H4 (Figure 7C), and trimethylated H3K4 (Figure 7D), were all up-regulated on the SPON2 promoter by oxLDL treatment. BRG1 knockdown significantly attenuated the accumulation of these active histone markers. On the contrary, oxLDL treatment resulted in a decrease in dimethyl H3K9, a histone marker typically found associated with repressed chromatin, on the SPON2 promoter, a trend which was reversed by BRG1 knockdown (Figure 7E). Similar observations with regard to characteristic changes of histone modifications on the SPON2 promoter were made in TNF-α treated endothelial cells (Figures 7F–J). Collectively, these data support a role for BRG1 in modulating chromatin structure to activate SPON2 transcription in response to pro-atherogenic stimuli.

## Discussion

Epigenetic regulation of gene expression is increasingly being recognized as a key process in the pathogenesis of atherosclerosis (Khyzha et al., 2017; Xu et al., 2018; Elia and Condorelli, 2019; Rizzacasa et al., 2019). Long considered a human pathology of chronic inflammation, atherogenesis is defined and programmed by the interplay between the vasculature and various immune cells. For instance, depletion of circulating macrophages by several different strategies attenuates atherosclerosis in experimental animals highlighting the critical role this population of immune cells play in atherogenesis (Ozaki et al., 2002; Sun et al., 2010; Bharath et al., 2015; Liu M. et al., 2019). Here we provide evidence to show that BRG1, a chromatin remodeling protein, activates transcription of Spondin 2 (SPON2) in vascular endothelial cells, which functions as a chemoattractant to promote macrophage migration (Figure 7K). There are several points worth stressing regarding this finding. First, our data echo previous reports that implicate BRG1 as an important regulator of atherogenesis. It has been shown previously that BRG1 interacts with NF-κB to activate the transcription of adhesion molecules and promote leukocyte adhesion to the vascular endothelium (Fang et al., 2013). Yuan et al. (2014) have reported that BRG1 over-expression activates the expression of pro-inflammatory mediators MMP2/MMP9 in vascular smooth muscle cells (VSMCs), induces apoptosis of VSMCs, and pivots VSMCs from a contractile phenotype to a synthetic phenotype, all of which contribute to plaque destabilization. In addition, a series of reports demonstrating that BRG1 regulates the identities of immune cells including macrophages (Ramirez-Carrozzi et al., 2006), B lymphocytes (Bossen et al., 2015), and T lymphocytes (Wurster and Pazin, 2008; De et al., 2011) suggesting that BRG1 may potentially influence the inflammatory makeup of the atherosclerotic plaque. Since small-molecule BRG1 inhibitors are already available (Wu et al., 2016), these results point to the possibility of exploiting these chemicals as an interventional approach against atherosclerosis. Second, we show here that *trans*-activation of SPON2 by BRG1 is accompanied by dynamic alterations of histone modifications on the SPON2 promoter. Consistent with our finding, a recent study has found that increased histone acetylation and H3K4 methylation and decreased H3K9 methylation are associated with severity of atherosclerosis in humans (Greissel et al., 2016). Whether the specific modifying enzymes are essential for SPON2 induction by pro-atherogenic stimuli to promote macrophage migration remains to be determined. We have previously demonstrated that the H3K9 di-demethylase KDM3A is a binding partner for BRG1 in endothelial cells (Zhang et al., 2018b). It has recently been shown that KDM3A promotes phenotypic modulation of VSMCs in diabetic rats (Chen et al., 2017), which is consistent with a potentially pro-atherogenic role for KDM3A. Third, the finding that endothelial-derived, BRG1-dependent SPON2 may dictate macrophage trafficking during atherogenesis is in line with an emerging role for BRG1 as a key transcriptional regulator of angiocrine signals. BRG1-mediated production and release of diffusive factors from vascular endothelial cells, including NO (Fish et al., 2010), endothelin (Weng et al., 2015), ROS (Li et al., 2018e), and CSF1 (Zhang et al., 2018b), can contribute to the pathogenesis of pulmonary hypertension, pathological cardiac hypertrophy, cardiac ischemia-reperfusion injury, and aortic aneurysm. This line of investigation would benefit from the profiling of BRG1-depedent endothelial secretome so that a more comprehensive role can be assigned to BRG1.

Our data support Egr-1 as the sequence-specific transcription factor responsible for recruiting BRG1 to the SPON2 promoter. A sea of evidence points to a pro-atherogenic role for Egr-1. It has been noted that Egr-1 deficiency, either global (Harja et al., 2004) or restricted to myeloid cells (Albrecht et al., 2010), protects the mice from atherosclerosis. Amelioration of atherosclerosis by Egr-1 deletion in mice is accompanied by a concomitant decrease in vascular inflammation, which could be attributed to down-regulation of such Egr-1 target genes as ICAM-1, VCAM-1, and IL-1β although no evidence is available to directly implicate Egr-1 in promoting macrophage homing to the plaque. A large body of evidence illustrates Egr-1 as a key regulator of endothelial dysfunction in the context of atherogenesis. For instance, Egr-1 can be activated by fluid shear stress (Khachigian et al., 1997; Schwachtgen et al., 1998) and oxidized phospholipids (Bochkov et al., 2002), two classic risk factors for atherosclerosis, in endothelial cells; activated Egr-1, in turn, functions to up-regulate the expression of pro-atherogenic genes (e.g., tissue factor). Further studies are warranted to determine whether endothelial cell conditional deletion of Egr-1 would be sufficient to retard macrophage infiltration and delay atherogenesis in mice.

Notably, SPON2 knockdown in endothelial cells did not alter the expression of adhesion molecules, suggesting that SPON2 may not regulate macrophage migration through mediating its interaction with endothelial cells (Supplementary Figure S4). Mounting evidence indicates that SPON2 may function as a ligand for cell-surface pattern recognition receptors to promote trafficking of immune cells. For instance, Jia et al. (2005) have reported that SPON2 stimulates the recruitment of macrophages and neutrophils to inflammatory foci by simultaneously binding to a group of integrin proteins. More recently, Liu Y. S. et al. (2019) and Zhang et al. (2018d) have independently reported that SPON2 can bind to integrin Mac-1 and integrin α4/β5, respectively, to promote innate immune response. Whether a similar mechanism accounts for SPON2-mediated macrophage migration in the context of atherosclerosis remains to be determined. Our data also demonstrate that SPON2 depletion did not alter the expression of several well-documented chemokines in endothelial cells (Supplementary Figure S5), arguing that SPON2 itself may be the predominant endothelial-derived and Egr-1/BRG1-stimulated chemoattractive cue to promote macrophage migration. Model animals harboring endothelial-specific SPON2 deletion should be exploited in future studies to verify whether SPON2 is indeed indispensable for macrophage recruitment to the atherosclerotic lesions *in vivo*.

Systemic (germline) SPON2 deletion in mice is associated with attenuation of atherosclerotic development, which is presumably due to impediment in foam cell formation (Zhang et al., 2018a). SPON2 expression is clearly detectable in endothelial cells but a functional role for SPON2 in this compartment remains elusive (Dreger et al., 2012; Pinto et al., 2018). Our data suggest that endothelial-derived SPON2 may play an important role in recruiting macrophages *in vitro*. Although the validity of this conclusion awaits further authentication in animal models, this newly identified role for SPON2 certainly renews the argument that SPON2 neutralization by targeting Egr-1/BRG1 may be a reasonable approach when devising interventional strategies against atherosclerosis.

## Data Availability Statement

The datasets generated for this study are available on request to the corresponding author.

## Ethics Statement

The animal study was reviewed and approved by Nanjing Medical University Ethics Committee on Humane Treatment of Experimental Animals.

## Author Contributions

MF and YX conceived the project. NL, SL, YZ, LY, and TW designed and performed the experiments, and analyzed the data. YX wrote the manuscript. YH, MF, and TW secured funding and provided supervision. All authors contributed to the article and approved the submitted version.

## Conflict of Interest

The authors declare that the research was conducted in the absence of any commercial or financial relationships that could be construed as a potential conflict of interest.
