# Corrigendum: Transcriptional Activation of Matricellular Protein Spondin2 (SPON2) by BRG1 in Vascular Endothelial Cells Promotes Macrophage Chemotaxis

**DOI:** 10.3389/fcell.2021.657085

**Published:** 2021-02-10

**Authors:** Nan Li, Shuai Liu, Yuanyuan Zhang, Liming Yu, Yanjiang Hu, Teng Wu, Mingming Fang, Yong Xu

**Affiliations:** ^1^Department of Cardiothoracic Surgery, Liyang People's Hospital, Liyang, China; ^2^Department of Clinical Medicine and Laboratory Center for Experimental Medicine, Jiangsu Health Vocational Institute, Nanjing, China; ^3^Key Laboratory of Targeted Intervention of Cardiovascular Disease and Collaborative Innovation Center for Cardiovascular Translational Medicine, Department of Pathophysiology, Nanjing Medical University, Nanjing, China; ^4^Institute of Biomedical Research, Liaocheng University, Liaocheng, China; ^5^Hainan Provincial Key Laboratory for Tropical Cardiovascular Diseases Research and Key Laboratory of Emergency and Trauma of Ministry of Education, Institute of Cardiovascular Research of the First Affiliated Hospital, Hainan Medical University, Haikou, China; ^6^Department of Cardiology, Kaifeng Central Hospital, Kaifeng, China

**Keywords:** transcriptional regulation, epigenetics, endothelial cells, macrophage, atherosclerosis

In the published article, there was an error in affiliation 6. Instead of “Department of Cardiology, Kaifeng People's Hospital, Kaifeng, China,” it should be “Department of Cardiology, Kaifeng Central Hospital, Kaifeng, China.”

The authors apologize for this error and state that this does not change the scientific conclusions of the article in any way. The original article has been updated.

