# Supplementary material for: Transcriptional Activation of Matricellular Protein Spondin2 (SPON2) by BRG1 in Vascular Endothelial Cells Promotes Macrophage Chemotaxis
Source: Front Cell Dev Biol. 2020 Aug 14;8:794. doi: 10.3389/fcell.2020.00794 (PMC7461951; doi:10.3389/fcell.2020.00794)
Supplement: Supplementary file 1 [file Table_1.DOCX]

**Li N et al: Transcriptional activation of matricellular protein Spondin2 (SPON2) by BRG1 in vascular endothelial cells promotes macrophagechemotaxis**

**Online supplementary material**

**Fig.S1:** 8-week male *Apoe*^-/-^ mice were fed a Western diet or a control diet for 12 weeks as described in Methods. Representative images of thoracic aorta stained with oil red O. Quantification was performed with Image Pro. N=6 mice for each group.

**Fig.S2:** 8-week male endothelial conditional BRG1 knockout mice in an *Apoe*^-/-^ background (*Smarca4*^f/f^;*Cdh5*-Cre;*Apoe*^-/-^) and the control mice (*Smarca4*^f/f^; *Apoe*^-/-^) were fed a Western diet for 12 weeks as described in the Methods. Representative images of thoracic aorta stained with oil red O. Quantification was performed with Image Pro. N=6 mice for each group.

**Fig.S3:** EAhy926 cells or HAECs were transfected with siRNAs targeting BRG1 or scrambled siRNA (SCR). BRG1 expression levels were examined by Western blotting.

**Fig.S4**: (**A**) EAhy926 cells were transfected with siRNAs targeting SPON2 or scrambled siRNA (SCR) followed by treatment with oxLDL (50μg/ml) for 24h. Expression levels of adhesion molecules were examined by qPCR. (**B**) EAhy926 cells were transfected with siRNAs targeting SPON2 or scrambled siRNA (SCR) followed by treatment with TNF-α (10ng/ml) for 24h. Expression levels of adhesion molecules were examined by qPCR. (**C**) EAhy926 cells were transfected with siRNAs targeting SPON2 or scrambled siRNA (SCR) followed by treatment with oxLDL (50μg/ml) or TNF-α (10ng/ml) for 24h. ChIP assays were performed with anti-BRG1.

**Fig.S5**: (**A**) EAhy926 cells were transfected with BRG1 and/or siRNA targeting SPON2 followed by treatment with oxLDL (50μg/ml) for 24h. Expression levels of chemokines were examined by qPCR. (**B**) EAhy926 cells were transfected with BRG1 and/or siRNA targeting SPON2 followed by treatment with TNF-α (10ng/ml) for 24h. Expression levels of chemokines were examined by qPCR.

**Fig.S6**: The human and the mouse SPON2 promoter sequence. The conserved Egr-1 motif is shown in bold and boxed. The numbers indicate position relative to the transcription start site.
